# A multi-country examination of the relationship between perfectionism and disordered eating: the indirect effect of obsessive beliefs and obsessive-compulsive symptoms

**DOI:** 10.1186/s40337-024-01030-y

**Published:** 2024-05-31

**Authors:** Feten Fekih-Romdhane, Susanna Pardini, Souheil Hallit, Caterina Novara, Anna Brytek-Matera

**Affiliations:** 1 The Tunisian Center of Early Intervention in Psychosis, Department of Psychiatry “Ibn Omrane”, Razi hospital, Manouba, 2010 Tunisia; 2 Faculty of Medicine of Tunis, Tunis El Manar University, Tunis, Tunisia; 3 Department of General Psychology, University of Padova, Padova, 35131 Italy; 4 School of Medicine and Medical Sciences, Holy Spirit University of Kaslik, P.O. Box 446, Jounieh, Lebanon; 5 Applied Science Research Center, Applied Science Private University, Amman, Jordan; 6 Eating Behavior Laboratory (EAT Lab), Institute of Psychology, University of Wrocław, Wrocław, 50-527 Poland

**Keywords:** Perfectionism, Disordered eating, Obsessive beliefs, Obsessive-compulsive disorder

## Abstract

**Background:**

Despite the extensive literature on the association between perfectionism and disordered eating (DE), only scant attention has been given to the underlying processes that may mediate this relationship. The present study aimed to contribute to existing literature by investigating the direct and indirect relations between perfectionism and DE through obsessive-compulsive disorder (OCD) symptoms and obsessive beliefs, among community adults from three different countries and cultural backgrounds (i.e. Poland, Italy and Lebanon).

**Methods:**

This is a cross-sectional study that was carried-out among 977 community adults (77.1% females, mean age: 21.94 ± 3.14 years) using the snowball sampling technique.

**Results:**

Obsessive-compulsive disorders (OCD) symptoms and obsessive beliefs had a partial indirect effect in the relationship between multidimensional perfectionism and disordered eating. Higher multidimensional perfectionism/obsessive beliefs were significantly associated with greater OCD symptoms and directly associated with higher DE scores. Finally, higher OCD symptoms were significantly linked to higher DE scores.

**Conclusion:**

The preliminary results suggest that it would be helpful for clinicians to routinely include measures of perfectionism, OCD and obsessive beliefs when dealing with individuals who present DE problems. In addition, results hold promise for the combined use of perfectionism and OCD interventions as a potentially beneficial treatment option for DE concerns.

**Supplementary Information:**

The online version contains supplementary material available at 10.1186/s40337-024-01030-y.

## Abstract (plain language summary)

Despite the extensive literature on the association between perfectionism and disordered eating (DE), only scant attention has been given to the underlying processes that may have an indirect effect in this relationship. Obsessive-compulsive disorders (OCD) symptoms and obsessive beliefs had a partial indirect effect in the relationship between multidimensional perfectionism and disordered eating. The preliminary results suggest that it would be helpful for clinicians to routinely include measures of perfectionism, OCD and obsessive beliefs when dealing with individuals who present disordered eating problems. In addition, results hold promise for the combined use of perfectionism and OCD interventions as a potentially beneficial treatment option for disordered eating concerns.

**Supplementary Information:**

The online version contains supplementary material available at 10.1186/s40337-024-01030-y.

## Introduction

Disordered eating (DE) pathology refers to a range of disturbed attitudes and behaviors related to eating patterns, shape and weight, which are less severe or occur less frequently compared to those required to meet the full criteria for the diagnosis of an eating disorder [1]. DE commonly occurs in the general population. Globally, meta-analyses reported pooled prevalence rates of DE of 22.36% among children and adolescents [2], 17–20% among university students [3, 4], and 8–22% among Western Asian people [5]. The proportion of affected people within the general population has been steadily increasing over the last decades, not only in Western society (e.g [6, 7]) but also in Middle Eastern contexts [8], which mirrors the sociocultural changes and economic developments in the region. DE has proven to result in long-term consequences on health in young adults, including higher body mass index, larger waist circumference, poor self-rated health, and increased psychological distress [9]. Despite these outcomes, the effectiveness of currently available treatments might be limited, leading researchers to call for an urgent need for novel treatment options and strategies to mitigate DE [10]. Elucidating the pathways that give rise to DE could pave the way for developing more effective prevention and intervention strategies. To date, the exact aetiology and underlying mechanisms that lead to DE remain unclear and poorly understood, with available evidence suggesting multifactorial causation involving a complex interaction of numerous genetics, environmental and psychological factors [11]. One potential suggested route through which DE may be developed and maintained is perfectionism [12].

### *The relationship between perfectionism and DE*

Perfectionism refers to a set of excessively unrealistic and high personal standards with overly self-critical evaluations of one’s behavior. It is most commonly conceptualized as a multidimensional construct that underlies different facets of perfectionism, including strivings for the pursuit of unrealistic goals, concerns for making mistakes and uncertainty regarding actions, and concerns for being negatively judged by others [13]. Recent findings of a cross-temporal meta-analysis involving multi-country samples of young adults pointed to a significant linear increase in prevalence estimates of perfectionism over the last three decades [14]. Perfectionism was found to be a transdiagnostic risk factor that plays a major role in a large array of different forms of psychopathology and maladaptive outcomes, including depression, anxiety disorders, obsessive-compulsive symptoms, obsessive beliefs, social phobia symptoms, psychological distress and suicidal ideation [12]. In addition, there is strong evidence from multiple systematic reviews and meta-analyses indicating that perfectionism represents an important causal factor contributing to disordered eating (DE) pathology, such as body dissatisfaction, dietary restraint, drive for thinness, thin-ideal internalisation [12], bulimia nervosa [15], binge eating [16], anorexia nervosa [17]. Extensive literature supports the relationship between perfectionism and DE in various populations, including children [18], adolescents [15, 19, 20], and adults [21]. In particular, perfectionism appears to be a robust predictor of both the emergence and persistence of DE, as it is found to precede the disease onset and to be present throughout the course and early stages of recovery [22]. In addition, a small but growing body of literature in both clinical and nonclinical populations suggests that treating perfectionism (e.g., through cognitive behavioral therapy) can be useful and effective in reducing DE symptomatology with large effect sizes, and in enhancing the outcome of affected individuals (for reviews and meta-analysis, see [23, 24]). Although there is extensive literature available supporting a positive association between perfectionism and DE, the underlying mechanisms behind this relationship remain unclear and unknown. For this reason, several scholars identified as an important direction for future research an investigation of the possible added role of potential covariates, such as obsessive-compulsive disorder (OCD) manifestations, to see if they exert a mediating or moderating effect on the relationship between perfectionism and DE [19].

### *OCD and obsessive beliefs as mediators*

Our indirect effect hypothesis is theoretically driven, given the previous evidence that obsessive-compulsive symptoms and beliefs are consistently regarded as a consequence of perfectionism [25, 26], and shown to be a significant modifiable risk factor for the development and exacerbation of DE [27, 28]. According to the DSM-5, OCD is recognized as a disorder encompassing the presence of obsessions (i.e., persistent and recurrent images, thoughts, or urges that are experienced as intrusive and unwanted) and/or compulsions (i.e., repetitive mental behaviors or acts that one feels driven to apply rigidly in response to obsessions) [29]. Obsessive beliefs (OB) as described by the Obsessive Compulsive Cognitions Working Group (OCCWG) [30] as sex belief domains that may lead to OCD, namely “responsibility” (i.e., the belief in one’s ability and obligation to prevent negative events), “importance of thoughts” (i.e., the simple occurrence of a thought signifies that it is meaningful and dangerous), “overestimation of threat” (i.e., overstated beliefs in the possibility and gravity of harm occurring), “intolerance of uncertainty” (i.e., the belief that ambiguity is intolerable and that it is necessary to be certain), and “control of thoughts” (i.e., the belief that it is necessary and feasible to control thoughts).

The role of perfectionism as an important predictor of OCD and its hypothesized involvement in the aetiology of OCD have been observed for decades [31]. Indeed, perfectionism is regarded as “a dispositional tendency” typically noticeable from childhood, and prospectively linked to later OCD symptoms [26]. Using latent growth curve modelling, a Belgian longitudinal study found that initial childhood perfectionism levels were significant positive predictors of OCD in adolescence, suggesting that perfectionism seem to be a vulnerability factor for developing OCD-related symptoms later in life [32]. Earlier research has, therefore, highlighted the value of baseline perfectionism for understanding subsequent differential occurrence of OCD [26, 32].

On the other hand, a meta-analysis of the literature revealed a current OCD prevalence of 15% and a lifetime OCD prevalence of 18% among patients diagnosed with a current primary eating disorder globally [33]. In addition to robust cross-sectional relationships, individuals with OCD experience high levels of eating pathology over time. For instance, a longitudinal study by Micali et al. [27] revealed that childhood OCD was related to a heightened risk of developing eating disorders later in life. Likewise, a US study by Buckner et al. [28] demonstrated a temporal link between OCD in adolescence and anorexia nervosa in adulthood over a 14-year follow-up period, after controlling for mood disorders and anxiety disorders. OB is also shown to increase the likelihood of the occurrence of DE. OB, such as intolerance of uncertainty, are suggested to represent both vulnerability and maintenance factors for DE [34].

### *Rationale of the present study*

This study was motivated by several considerations. Despite the extensive literature on the relationship between perfectionism and DE, only scant attention has been given to the underlying processes that may mediate this relationship. In addition, researchers observed that most of the existing research in this area predominantly focused on specific populations (such as young, female, and Caucasian), and highlighted the strong need for further studies on perfectionism and DE to be conducted with more diverse populations (i.e., different age/sex/ethnic groups, cultures, countries) [24]. Indeed, socio-cultural and religious influences have been proposed to impact the prevalence and manifestation of DE within and across communities [35, 36]. Prior findings have also suggested that perfectionism is culturally dependent [37], with Middle Eastern young adults found to endorse greater parental expectations and self-oriented perfectionism than their US counterparts [38].

In this context, our study aimed to contribute to the body of knowledge by investigating the direct and indirect relations between perfectionism and DE through OCD and obsessive beliefs, among community adults from three different countries and cultural backgrounds (i.e. Poland, Italy and Lebanon). It is hypothesized that obsessive-compulsive symptoms and obsessive beliefs will have an indirect effect in the association between perfectionism and DE.

## Methods

### Study design

This study has a cross-sectional design, and was carried out in three different countries (i.e., Poland (Silesian region and Upper Silesia), Lebanon (all governorates), Italy (northern Italy), and involved a total of 977 adult participants. In order to be eligible, participants need to be aged 18 years and over, and be sampled from the general population of each country [39]. To reach a wider range of participants, the snowball sampling technique was employed. “Recruitment took place during university lessons; specifically, students were given a short general presentation of the project and invited to participate in the research. Individuals had to confirm their participation via email. Through creating an individual account, managed and monitored by the investigators, participants were sent a Google form link via email. Participants completed the questionnaires in a single online session; firstly, signing the informed consent form and filling in the personal datasheet. Then, a battery of counterbalanced self-report questionnaires was administered. A numerical code corresponded, accompanied with the informed consent form, tests, and the personal datasheet. Protocols with at least 10% of the answers omitted have been excluded” [39].

### Measures

Data was gathered using a self-report questionnaire composed of two parts: (1) a first part containing sociodemographic information (i.e., sex, country of origin, tobacco and alcohol use), and (2) a second part containing four measurement instruments that assess psychological functioning and eating behaviors. The questionnaire was administered in Polish for Polish participants, in Italian for Italian participants, and in English for Lebanese participants.

#### The eating attitudes test (EAT-26)

This is a 26-item measure assessing levels of disordered eating among participants. Each item is scored on a 6-point Likert scale (Always - Usually - Often - Sometimes - Rarely – Never), with greater scores indicating more severe disordered eating symptoms [40, 41], with the scale validated in Italian [42] and Polish [43] (Cronbach’s α = 0.92 for the total sample in this study).

#### The obsessive-compulsive inventory-revised (OCI-R)

The OCI-R [37] is a shortened version of the Obsessive-Compulsive Inventory [38]. It is a self-report scale composed of 18 items. It assesses the severity of OCD symptoms through the following six dimensions: neutralizing, hoarding, obsessing, ordering, checking, and washing. Items are scored on a five-point Likert scale ranging from 0 (not at all) to 4 (extremely). Greater scores indicate higher OCD levels. The OCI-R was used in its validated Italian [40], Polish [39] and English [37] versions for participants from Italy, Poland and Lebanon respectively (α = 0.92 for the total sample in this study).

#### The obsessive beliefs questionnaire-44 (OBQ-44)

The OBQ-44 is a self-report measure designed to evaluate domains related to OCD. The scale is composed of 44 divided into three subscales: (1) beliefs about perfectionism and intolerance of certainty (12 items; i.e. beliefs that imperfection and mistakes cannot be tolerated), (2) importance and control of thoughts (16 items; i.e. beliefs that it is possible and necessary to control thoughts), and (3) responsibility and overestimation of threat (16 items; i.e. beliefs that one is able and especially obligated to prevent subjectively important negative events). Each item is rated on a seven-point scale ranging from 1 (“disagree very much”) to 7 (“agree very much”). Total scores range from 44 to 308, with more elevated scores reflecting greater OCD-related beliefs [41]. As the OBQ-44 was not validated in Polish and Italian, the forward and backward translation process following international recommendations was adopted to ensure that the measure is linguistically and culturally appropriate for the target population [44]. Regarding the Polish translation, the OBQ was translated from English to Polish using a standard forward–backward translation procedure. The English version of the OBQ was first translated into Polish (by two translators who independently translated the same questionnaire) and then back-translated into English (by two independent native English speakers without reference to the English original). The Italian version was used in the present study [45].

The initial and translated English versions were compared to detect and later eliminate any inconsistencies [46, 47]. A pilot study was conducted on 30 persons before the start of the official data collection to make sure all questions are well understood; no changes were done consequently [48] (α = 0.97 for the total sample in this study).

#### The multidimensional perfectionism scale (MPS)

The Multidimensional Perfectionism Scale (MPS) is a commonly used inventory that measures different aspects of perfectionism. It consists of 35 questions that assess four sub-scales of perfectionism, which are: concern over mistakes and doubts about actions, excessive concern with parents’ expectations and evaluation, excessively high personal standards, and concern with precision, order, and organization. The results of the inventory provide both a Total Perfectionism score and scores for each of the four subscales. The MPS is a useful tool for identifying the various dimensions of perfectionism and can be helpful in both research and clinical settings [39, 40]. The MPS has been translated and validated in Polish [44], and Italian [45] (α = 0.91 for the total sample in this study).

#### Statistical analysis

The SPSS software version 23 (IBM Corp., Armonk, NY, USA) was employed to analyze data. Scales’ reliability values were examined using Cronbach’s alpha values. The kurtosis and skewness of the EAT-26 total score was considered normally distributed since they varied between − 1 and + 1 [49]. The ANOVA test was used to compare three or more means, the Student’s t-test to compare two means and the z test statistic was used to compare the Pearson correlation coefficients of two continuous variables. The mediation analysis was conducted using PROCESS MACRO (an SPSS add-on) v.3.4 model 4; four pathways derived from this analysis: pathway A from the independent variable to the mediator, pathway B from the mediator to the dependent variable, Pathways C and C’ indicating the total and direct effects from the independent to the dependent variable. Results adjusted over variables that showed a *p* < .25 in the bivariate analysis. We considered the mediation analysis to be significant if the Boot Confidence Interval did not pass by zero. *P* < .05 was considered significant.

## Results

A total of 977 adults from the general population participated in this study (77.1% females, mean age: 21.94 ± 3.14 years). Other sociodemographic information is illustrated in Table 1.

**Table 1:** Sociodemographic and other characteristics of the participants (*n* = 977)

	Total (*n* = 977)	Poland (*n* = 283)	Italy (*n* = 319)	Lebanon (*n* = 375)
Variable	*n* (%)	*n* (%)	*n* (%)	*n* (%)
Sex				
Male	224 (22.9%)	49 (17.3%)	66 (20.7%)	109 (29.1%)
Female	753 (77.1%)	234 (82.7%)	253 (79.3%)	266 (70.9%)
Smoking				
Yes	211 (21.6%)	69 (24.5%)	72 (22.6%)	70 (18.7%)
No	765 (78.4%)	213 (75.5%)	247 (77.4%)	305 (81.3%)
Alcohol drinking				
Yes	583 (59.7%)	194 (68.8%)	224 (70.2%)	165 (44.0%)
No	393 (40.3%)	88 (31.2%)	95 (29.8%)	210 (56.0%)
	**Mean ± SD**	**Mean ± SD**	**Mean ± SD**	**Mean ± SD**
Age, years	21.94 ± 3.14	22.37 ± 2.35	21.91 ± 2.08	21.63 ± 4.19
Body Mass Index (kg/m^2^)	22.38 ± 3.97	22.73 ± 4.31	21.41 ± 3.10	22.93 ± 4.21
Disordered eating (Eating Attitude Test-26)	10.83 ± 12.48	8.43 ± 9.16	5.64 ± 7.65	16.83 ± 15.13
Multidimensional perfectionism	101.88 ± 21.33	100.45 ± 22.11	98.76 ± 20.06	105.26 ± 21.79
Obsessive-compulsive symptoms	19.21 ± 13.50	17.66 ± 13.67	13.13 ± 8.62	25.40 ± 14.21
Obsessive beliefs	148.08 ± 47.58	142.84 ± 47.03	135.20 ± 40.63	166.83 ± 46.61

### Bivariate analysis

Higher DE scores were found in Lebanese compared to Polish and Italians, and in participants who did not drink alcohol (Table 2). Finally, older age was significantly associated with lower DE scores, whereas higher BMI, multidimensional perfectionism, obsessive beliefs and obsessive-compulsive symptoms were significantly associated with higher EAT scores (Table 3). The results of the bivariate analysis of factors associated with multidimensional perfectionism, obsessive-compulsive symptoms and obsessive beliefs, are summarized in Supplementary Table 1.

**Table 2:** Bivariate analysis of factors associated with disordered eating

	Mean ± SD	*p*	t / F	df
Sex		0.989	− 0.01	959
Male	10.74 ± 12.06			
Female	10.76 ± 12.64			
Country		**< 0.001**	91.33	2, 958
Poland	8.54 ± 9.31			
Italy	5.48 ± 7.41			
Lebanon	16.83 ± 15.13			
Smoking		0.604	0.52	959
No	10.86 ± 12.59			
Yes	10.36 ± 12.21			
Alcohol drinking		**< 0.001**	5.35	959
No	13.32 ± 14.12			
Yes	8.99 ± 10.93			

Numbers in bold indicate significant *p* values

**Table 3:** Pearson correlation matrix

	1	2	3	4	5	6
1. Disordered eating	1					
2. Age	− 0.10**	1				
3. BMI	0.11**	0.06	1			
4. Obsessive beliefs	0.32***	− 0.14***	0.04	1		
5. Multidimensional perfectionism	0.27***	− 0.07*	0.02	0.58***	1	
6. Obsessive-compulsive symptoms	0.43***	− 0.12***	0.05	0.55***	0.45***	1

*p < .05; **p < .01; ***p < .001

### Indirect effect

**Fig. 1:**
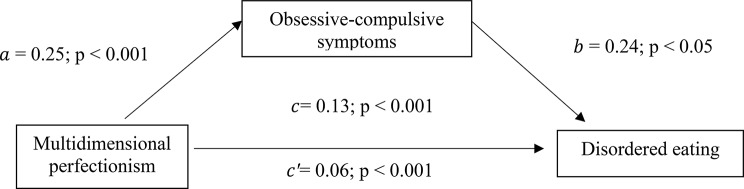
(a) Relation between multidimensional perfectionism and obsessive-compulsive symptoms (R^2^ = .324); (b) Relation between obsessive-compulsive symptoms and disordered eating (R^2^ = .273); (c) Total effect of multidimensional perfectionism on disordered eating (R^2^ = .221); (c’) Direct effect of multidimensional perfectionism on disordered eating. Numbers are displayed as regression coefficients with their *p* values respectively

**Fig. 2:**
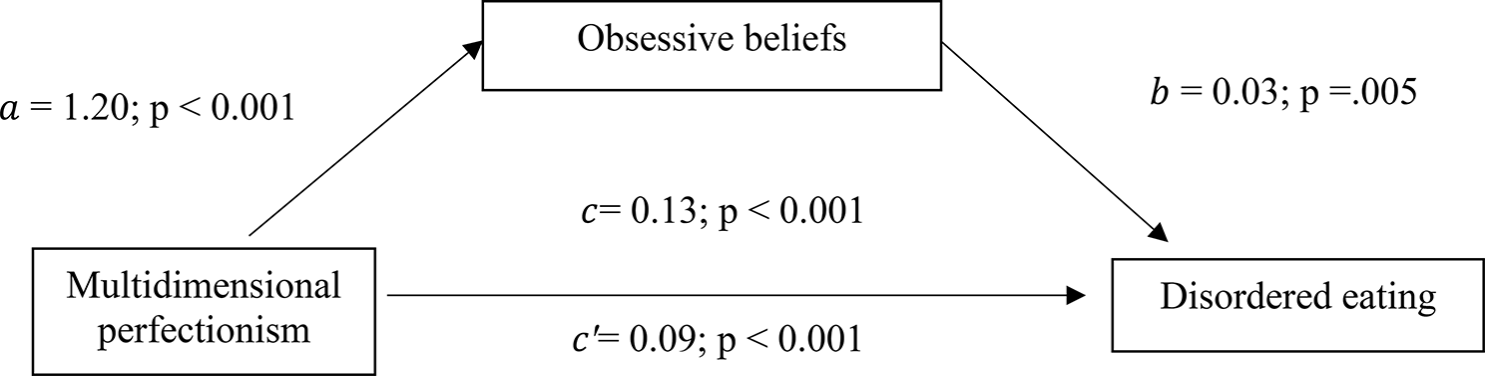
(a) Relation between multidimensional perfectionism and obsessive beliefs (R^2^ = .418); (b) Relation between obsessive beliefs and disordered eating (R^2^ = .228); (c) Total effect of multidimensional perfectionism on disordered eating (R^2^ = .221); (c’) Direct effect of multidimensional perfectionism on disordered eating. Numbers are displayed as regression coefficients (standard error). ***p < .001

The results of the analysis were adjusted over the following variables: country, BMI, age and alcohol drinking. Obsessive-compulsive symptoms (indirect effect: Beta = 0.07; Boot SE = 0.01; Boot CI 0.05 − 0.09) and obsessive beliefs (indirect effect: Beta = 0.03; Boot SE = 0.01; Boot CI 0.01 − 0.06) had a partial indirect effect in the association between multidimensional perfectionism and DE. Higher multidimensional perfectionism/obsessive beliefs were significantly associated with higher OCD and directly associated with higher DE scores. Finally, higher OCD was significantly associated with higher DE scores (Tables 4 and 5; Figs. 1 and 2).

**Table 4:** Analysis taking the indirect effect of obsessive-compulsive symptoms between multidimensional perfectionism and disordered eating

	Beta	t	*p*	95% CI
Model 1: Multidimensional perfectionism on obsessive-compulsive symptoms (*R*^2^ = 0.324)				
Multidimensional perfectionism	0.25	0.02	**< 0.001**	0.22 − 0.29
Italy vs. Poland*	-4.50	-4.62	**< 0.001**	-6.40 -2.59
Lebanon vs. Poland*	6.36	6.66	**< 0.001**	4.49–8.24
BMI	0.01	0.08	0.937	− 0.18 − 0.20
Age	− 0.32	-2.73	**0.006**	− 0.55 - − 0.09
Alcohol drinking (yes vs. no*)	1.07	1.39	0.164	− 0.44–2.58
**Model 2: Obsessive-compulsive symptoms on disordered eating (R**^**2**^ **= 0.273)**				
Multidimensional perfectionism	0.06	3.32	**< 0.001**	0.03 − 0.10
Obsessive-compulsive symptoms	0.26	8.07	**< 0.001**	0.19 − 0.32
Italy vs. Poland*	-1.67	-1.76	0.079	-3.53 − 0.20
Lebanon vs. Poland*	5.13	5.43	**< 0.001**	3.28–6.99
BMI	0.27	2.88	**0.004**	0.08 − 0.45
Age	− 0.18	-1.55	0.121	− 0.40 − 0.05
Alcohol drinking (yes vs. no*)	-2.08	-2.79	**0.005**	-3.54 - − 0.62
**Model 3: Multidimensional perfectionism on disordered eating (R**^**2**^ **= 0.221)**				
Multidimensional perfectionism	0.13	7.32	**< 0.001**	0.09 − 0.16
Italy vs. Poland*	-2.82	-2.90	**0.004**	-4.73 - − 0.92
Lebanon vs. Poland*	6.77	7.09	**< 0.001**	4.89–8.64
BMI	0.27	2.80	**0.005**	0.08 − 0.46
Age	− 0.26	-2.20	**0.027**	− 0.49 - − 0.03
Alcohol drinking (yes vs. no*)	-1.80	-2.34	**0.019**	-3.31 - − 0.29

Numbers in bold indicate significant *p* values

**Table 5:** Analysis taking the indirect effect of obsessive beliefs between multidimensional perfectionism and Disordered Eating

	Beta	t	*p*	95% CI
**Model 1: Multidimensional perfectionism on obsessive beliefs (R** ^**2**^ ** = 0.418)**				
Multidimensional perfectionism	1.20	21.28	**< 0.001**	1.09–1.32
Italy vs. Poland*	-10.71	-3.38	**< 0.001**	-16.93 - -4.50
Lebanon vs. Poland*	17.82	5.72	**< 0.001**	11.71–23.94
BMI	0.19	0.60	0.549	− 0.43 − 0.80
Age	-1.23	-3.21	**0.001**	-1.98 - − 0.48
Alcohol drinking (yes vs. no*)	-1.11	− 0.44	0.658	-6.03–3.82
**Model 2: Obsessive beliefs on Disordered Eating (R**^**2**^ **= 0.228)**				
Multidimensional perfectionism	0.09	4.47	**< 0.001**	0.05 − 0.14
Obsessive-compulsive symptoms	0.03	2.70	**0.007**	0.01 − 0.05
Italy vs. Poland*	-2.53	-2.60	**0.009**	-4.44 - − 0.62
Lebanon vs. Poland*	6.28	6.49	**< 0.001**	4.38–8.18
BMI	0.26	2.76	**0.006**	0.08 − 0.45
Age	− 0.23	-1.92	0.055	− 0.46 − 0.01
Alcohol drinking (yes vs. no*)	-1.77	-2.31	**0.021**	-3.28 - − 0.27
**Model 3: Multidimensional perfectionism on Disordered Eating (R**^**2**^ **= 0.221)**				
Multidimensional perfectionism	0.13	7.32	**< 0.001**	0.09 − 0.16
Italy vs. Poland*	-2.82	-2.90	**0.004**	-4.73 - − 0.92
Lebanon vs. Poland*	6.77	7.09	**< 0.001**	4.89–8.64
BMI	0.27	2.80	**0.005**	0.08 − 0.46
Age	− 0.26	-2.20	**0.027**	− 0.49 - − 0.03
Alcohol drinking (yes vs. no*)	-1.80	-2.34	**0.019**	-3.31 - − 0.29

Numbers in bold indicate significant *p* values

## Discussion

The present study proposes to delve into the mechanisms underlying the relationship perfectionism-DE, by exploring, for the first time, the indirect role of OCD and obsessive beliefs among community adults originating from different Western (Italy and Poland) and non-Western (Lebanon) cultural contexts. The study hypothesis was partially supported, as both factors (obsessive-compulsive symptoms and obsessive beliefs) acted as having a partial indirect effect between perfectionism and DE after controlling for main confounders (including country of origin). These results provide important theoretical and practical implications that will be discussed later.

There were no significant differences across males and females for ED scores in our sample. Findings in this area are inconsistent. Although it is commonly accepted that females would be at greater risk for screen-based DE around the globe [3], there is increasing evidence from more recent studies suggesting no significant sex difference in patterns of DE (e.g [50–53]). A recent systematic review revealed that both males and females were at a comparable risk of DE symptoms, and noted trends of increasing DE symptoms reporting among males across time [54]. Furthermore, Italian participants showed the lowest DE scores, followed by Polish then Lebanese participants. These findings are consistent with the results of a recent systematic review and metanalysis which showed that individuals from non-Western cultures exhibited a statistically higher pooled prevalence rate of DE symptoms than those from Western cultures (20.97% versus 12.98%, *p* = .01) [54].

As for direct effects, significant positive pathways between multidimensional perfectionism levels and DE (as measured using the EAT-26) were observed. These results provide additional support to previous evidence from recent systematic reviews suggesting that perfectionistic individuals experience an increased risk of DE pathology [12, 15–17]. The close positive connection between perfectionism and DE was found across multiple stages of the lifespan, including in adulthood [21]. In addition, perfectionism was identified as a key factor contributing to both the development and maintenance of DE [22]. Establishing a significant link between perfectionism and DE, both regarded as culture-and country-specific concepts [36, 37], even after controlling for country, suggests that the relationship between the two entities could be universal.

Regarding indirect effects, and as expected, the effects of both obsessive-compulsive symptoms and obsessive beliefs were statistically significant. The models tested were theoretically based on previous empirical research indicating, on the one hand, that perfectionism is related to OCD and obsessive beliefs [26, 32], and, on the other, that the latter are correlated with DE [27, 28, 34]. Perfectionism is regarded as “a viable candidate trait antecedent” involved in the clinical manifestation of subsequent OCD [32], and high prevalence rates of OCD are commonly reported in clinical eating disorder populations [33, 55]. These results suggest that early interventions targeting the indirect pathway through OCD and OB could be a sufficient and effective treatment strategy to decrease DE-related symptoms for DE. However, caution is required when interpreting our findings, given that they rely on cross-sectional data and that some previous evidence pointed to a bidirectional longitudinal association between OCD and DE [56]. As such, future research testing alternative mediation models (e.g., with OCD as the dependent variable and DE as a mediator) is warranted. Furthermore, it is of note that more than a half of participants (59.7%) reported being alcohol consumers, which might have affected findings, as alcohol consumption may vary across different perfectionism traits dimensions [57], often overlap with OCD symptoms [57], and commonly co-occur with DE [58, 59]. However, it needs to be highlighted that to minimize the confounding effect, the presence of alcohol consumption was controlled for in all models.

### Implications and future perspectives

Our study offers insights into how perfectionism, OCD symptoms and OB interact in people with DE; thus, paving the way for a more nuanced understanding of the relationship between perfectionism and ED. Findings have potential clinical implications for the targeted prevention and management of at-risk populations who experience DE in community settings. The significant indirect effect findings suggest that OCD and OB may be considered to portray potential DE outcomes, and can constitute practical constructs for alerting clinical experts when they encounter highly perfectionistic individuals with eating pathology in their clinical practice. Therefore, our preliminary findings hold promise for the combined use of perfectionism and OCD interventions as a potentially beneficial treatment option for DE concerns.

As for research perspectives, future studies are warranted to explore the indirect effects of OCD and obsessive beliefs in the association between perfectionism and DE during childhood and adolescence. In addition, there is a need to examine the role of other potential mediators/moderators in this relationship, such as internalizing psychopathology (i.e., anxiety and depression) [60, 61]. Moreover, since a relationship between other strictly related eating-disorder constructs, such as Orthorexia Nervosa, and perfectionism is highlighted [62], future studies should consider extending the investigation of the role of constructs related to eating habits in mediating or moderating the relationship between DE and perfectionism.

Finally, empirically demonstrating a significant direct and indirect association between perfectionism and DE in a sample of people from three different countries and cultures is meaningful, because it might suggest the universality of these relationships. Nevertheless, the interpretation of ‘universality’ could be limited in that it is a result of only three countries. This suggestion still needs to be confirmed by investigating larger samples from various cultures and countries.

### Study limitations

There were some limitations in this study, as in every research. First, self-report measurement instruments were used to assess all the study variables; therefore, responses may be affected by considerations of social desirability. Second, as the directionality of associations could not be assessed due to the cross-sectional design, more research is needed to shed light on the causal relationships linking perfectionism, OCD and DE together. Third, the recruitment relied on the snowball sampling technique, which may limit the generalization of findings to a certain extent; the sample may be influenced by the characteristics, preferences and opinions of the initial participants and their referrals. The sample was taken from a non-clinical setting; future studies are recommended to compare our results from the ones of clinical patients. Finally, the OBQ-44 scale is not validated in Italian and Polish; therefore, results should be interpreted with caution.

## Conclusion

In sum, and despite these limitations, this study contributes to research by showing significant direct and indirect associations between perfectionism and DE symptoms through OCD and obsessive beliefs in community adults from three different countries and cultures (i.e., two European and one Middle Eastern). These preliminary results emphasize the importance of targeting OCD symptoms and obsessive beliefs in highly perfectionist individuals who endorse DE. It is also cautiously recommended that clinicians systematically assess the presence of perfectionism, OCD and obsessive beliefs in people who experience DE using practical self-report measures. Early detection of these factors might help prevent the progression of DE into more severe conditions and worse outcomes. However, future longitudinal studies with more representative samples from diverse countries and using valid measures are warranted to confirm the present findings and draw generalizable conclusions about the pathways linking perfectionism, OCD, OB, and DE.

### Electronic supplementary material

Below is the link to the electronic supplementary material.

Supplementary Material 1

## Data Availability

The datasets generated and/or analyzed during the current study are not publicly available but are available from the corresponding author on reasonable request.
