# Supplementary material for: A multi-country examination of the relationship between perfectionism and disordered eating: the indirect effect of obsessive beliefs and obsessive-compulsive symptoms
Source: J Eat Disord. 2024 May 31;12:69. doi: 10.1186/s40337-024-01030-y (PMC11143671; doi:10.1186/s40337-024-01030-y)
Supplement: Supplementary file 1 — Supplementary Material 1 [file 40337_2024_1030_MOESM1_ESM.docx]

| **Supplementary Table 1. Bivariate analysis of factors associated with eating attitudes, multidimensional perfectionism, obsessive-compulsive symptoms and obsessive beliefs.** | | | | | | | | | | | | |
| --- | --- | --- | --- | --- | --- | --- | --- | --- | --- | --- | --- | --- |
|  | **Multidimensional perfectionism** | | | | **Obsessive-compulsive symptoms** | | | | **Obsessive beliefs** | | | |
|  | **Mean ± SD** | ***p*** | ***t /* F** | **df** | **Mean ± SD** | ***p*** | ***t /* F** | **df** | **Mean ± SD** | ***p*** | ***t /* F** | **df** |
| Sex |  | .081 | 1.75 | 923 |  | .166 | 1.39 | 945 |  | **.048** | 1.98 | 924 |
| Male | 104.05 ± 20.02 |  |  |  | 20.31 ± 13.95 |  |  |  | 155.16 ± 43.26 |  |  |  |
| Female | 101.14 ± 21.86 |  |  |  | 18.86 ± 13.39 |  |  |  | 148.33 ± 47.89 |  |  |  |
| Country |  | <.001 | 8.51 | 2, 922 |  | <.001 | 86.22 | 2, 944 |  | <.001 | 46.15 | 2, 923 |
| Poland | 100.20 ± 22.26 |  |  |  | 17.61 ± 13.61 |  |  |  | 141.94 ± 47.46 |  |  |  |
| Italy | 98.92 ± 19.92 |  |  |  | 13.12 ± 8.65 |  |  |  | 135.80 ± 40.31 |  |  |  |
| Lebanon | 105.26 ± 21.79 |  |  |  | 25.40 ± 14.21 |  |  |  | 166.83 ± 46.61 |  |  |  |
| Smoking |  | .074 | 1.79 | 923 |  | .971 | -.04 | 945 |  | .361 | .91 | 924 |
| No | 102.48 ± 21.70 |  |  |  | 19.19 ± 13.59 |  |  |  | 150.67 ± 46.46 |  |  |  |
| Yes | 99.43 ± 20.47 |  |  |  | 19.23 ± 13.32 |  |  |  | 147.26 ± 48.56 |  |  |  |
| Alcohol drinking |  | .147 | 1.45 | 923 |  | **.028** | 2.21 | 945 |  | **.006** | 2.76 | 924 |
| No | 103.03 ± 22.40 |  |  |  | 20.39 ± 14.28 |  |  |  | 154.96 ± 48.56 |  |  |  |
| Yes | 100.95 ± 20.76 |  |  |  | 18.38 ± 12.93 |  |  |  | 146.36 ± 45.43 |  |  |  |

Numbers in bold indicate significant p values.
